# Cerebrovascular Reactivity Assessed by Breath-Hold Functional MRI in Patients with Neurological Post-COVID-19 Syndrome—A Pilot Study

**DOI:** 10.3390/neurolint16050075

**Published:** 2024-09-09

**Authors:** Leonie Zerweck, Uwe Klose, Annerose Mengel, Tobias Hoheisel, Melinda Eikemeier, Vivien Richter, Natalie Sophie Joos, Ulrike Ernemann, Benjamin Bender, Till-Karsten Hauser

**Affiliations:** 1 Department of Diagnostic and Interventional Neuroradiology, University Hospital Tuebingen, 72076 Tuebingen, Germanybenjamin.bender@med.uni-tuebingen.de (B.B.); till-karsten.hauser@med.uni-tuebingen.de (T.-K.H.); 2 Department of Neurology and Stroke, University Hospital Tuebingen, 72076 Tuebingen, Germany; 3 Department of Traumatology and Reconstructive Surgery, BG Trauma Center Tuebingen, 72076 Tuebingen, Germany; 4 Division of Infectious Diseases, Department of Internal Medicine I, University Hospital Tuebingen, 72076 Tuebingen, Germany; 5 Department of Diagnostic and Interventional Radiology, University Hospital Tuebingen, 72076 Tuebingen, Germany

**Keywords:** post-COVID-syndrome, SARS-CoV-2, cerebrovascular reactivity, breath-hold functional MRI

## Abstract

Endothelial dysfunction represents a potential pathomechanism of neurological post-COVID-19 syndrome (PCS). A recent study demonstrated reduced cerebrovascular reactivity (CVR) in patients with PCS. The aim of this pilot study was to prospectively assess CVR in patients with PCS using breath-hold functional MRI (bh-fMRI). Fourteen patients with neurological PCS and leading symptoms of fatigue/memory issues/concentration disorder (PCS_fmc_), 11 patients with PCS and leading symptoms of myopathy/neuropathy (PCS_mn_), and 17 healthy controls underwent bh-fMRI. Signal change and time to peak (TTP) were assessed globally and in seven regions of interest and compared between the subgroups using one-way ANCOVA adjusting for age, time since infection, Fazekas score, and sex. No significant differences were observed. In PCS patients, the global CVR exhibited a slight, non-significant tendency to be lower compared to healthy controls (PCS_fmc_: 0.78 ± 0.11%, PCS_mn_: 0.84 ± 0.10% and 0.87 ± 0.07%). There was a non-significant trend towards lower global TTP values in the PCS subgroups than in the control group (PCS_fmc_: 26.41 ± 1.39 s, PCS_mn_: 26.32 ± 1.36 s versus 29.52 ± 0.93 s). Endothelial dysfunction does not seem to be the sole pathomechanism of neurological symptoms in PCS. Further studies in larger cohorts are required.

## 1. Introduction

The World Health Organization (WHO) defines post-COVID-19 syndrome (PCS) as a constellation of symptoms that occur more than 12 weeks after acute infection with SARS-CoV-2, persist for at least 2 months, which cannot be explained by an alternative etiology [1]. The clinical course may be persistent, relapsing, or fluctuating [1].

Symptoms vary widely and can sometimes lead to considerable limitations in daily life and even to incapacity to work [2,3]. The neurological symptoms associated with PCS are also highly variable and include exemplarily fatigue (37%), brain fog (32%), memory issues (28%), attention disorder (22%), myalgia (17%), anosmia (12%), dysgeusia (10%), and headache (15%) [4]. 

A heterogeneous and multifactorial pathogenesis of neurological symptoms are assumed [5,6]. Several possible mechanisms that may occur simultaneously are discussed [5,6] as follows: (i) direct neurological invasion and injury by the virus [5,7,8], (ii) para-infectious autoimmune responses directed against the central nervous system (CNS) [5], (iii) toxic effects of severe systemic COVID-19 disease on the CNS [5,9] and (iv) persistent endothelial dysfunction and COVID-19-associated coagulopathy [2,5,10,11,12,13]. In large autopsy studies, endothelial dysfunction was detected as endotheliitis in the pons, the thalami, juxta-cortically, and in deep white matter, identified by microbleeds, petechial hemorrhages within vessel walls, and perivascular infiltration of T cells and macrophages [5,14]. 

The vascular endothelium, as an essential part of the neurovascular unit, is the main regulator of cerebrovascular reactivity (CVR) [15], which is defined as the change in cerebral blood flow in response to a vasoactive stimulus [15,16,17]. In a recent study, a significantly lower whole-brain CVR in patients with previous SARS-CoV-2 infection than in never-before-infected participants was described, whereby the CVR was lower in patients with than without neurological PCS [15]. The CVR was measured using arterial spin labeling (ASL) perfusion imaging with acetazolamide stimulus [15]. Several studies using transcranial color Doppler (TCCD) and a breath-holding test also indicated a reduced CVR in patients with acute SARS-CoV-2 infection and post-SARS-CoV-2 conditions [12,18,19,20], although contradictory findings exist [21].

Hypercapnia-triggered functional magnetic resonance imaging (fMRI) is another option to assess CVR, eliminating the need for intravenous acetazolamide stimulation [16,22,23,24]. The increased arterial partial pressure of CO_2_ (PaCO_2_) induces cerebral vasodilation and increases cerebral blood flow [6,16]. The resulting altered ratio of paramagnetic deoxyhemoglobin to diamagnetic oxyhemoglobin in venules leads to changes in magnetic susceptibility and an increase in the blood-oxygen-level-dependent (BOLD) signal [25]. One highly available method to achieve hypercapnia in order to estimate CVR without the need for CO_2_ inhalation systems are breath-hold (bh) periods [8,13,16,23,24,25]. Bh-fMRI is highly available and has no possible side-effects and risks, such as the injection of acetazolamide, and can, therefore, be used safely in patients and healthy subjects [13]. As an advantage over TCCD, it provides a high spatial resolution and allows comparisons within any region of interest (ROI) after the normalization of the data set [13]. To date, only a few studies investigated CVR in PCS patients with specific neurological symptoms, and only Callen et al. used MRI [6].

The aim of this pilot study was to investigate whether CVR differences can be detected by bh-fMRI in patients with PCS and leading symptoms of fatigue/memory issues/concentration disorder (PCS_fmc_) or in patients with PCS and leading symptoms of myopathy/neuropathy (PCS_mn_), and persons previously infected with SARS-CoV-2 without PCS.

The second aim was to investigate if there is a correlation between global CVR and white matter changes in patients with PCS.

## 2. Materials and Methods

A prospective bh-fMRI study of patients with neurological PCS and persons previously infected with SARS-CoV-2 without PCS was performed. The study was approved by the local ethics committee. Written informed consent was obtained from all participants.

### 2.1. Participants

#### 2.1.1. Patients with PCS

Patients with PCS and concomitant neurological symptoms were recruited by the treating physicians during the PCS consultation hours or PCS physical rehabilitation. The recruitment of the participants began on 1 December 2022 and was completed on 30 June 2024. Inclusion criteria were an age of 18–50 years and PCS diagnosed according to the WHO criteria presenting with neurological symptoms (either leading symptoms of fatigue/memory issues/concentration disorder or leading symptoms of myopathy/neuropathy). Fatigue was defined as ≥36 points on the Fatigue Severity Scale (FSS) [26]. Memory issues were defined as ≤26 points (≤25 if ≤12 years of education) on the Montreal Cognitive Assessment (MoCA) screening [27] or ≥ 2 SD below the age- and education-corrected score on the Symbol Digit Modalities Test (SDMT) [28]. Concentration disorder was defined by a time to completion ≥2 SD above the age- and education-corrected score on the Trail Making Test-B (TMT-B) [29]. Neuropathy was diagnosed by electrophysiological examination with reduced nerve conduction velocity (NLG) ≥ 30% below the norm for age in two nerves [30] or with neuropathologically proven intraepidermal nerve fiber density (IENFD) (age- and sex-corrected) below the 5th quantile by skin biopsy [31]. Myopathy was determined by Quantitative Motor (Q-Motor) testing with ≥2 SDs of the Grip Force Assessment (QGFA) and of the Involuntary Movement Assessment (QIMA) compared to healthy controls [32]. If patients showed symptoms in both subgroups, group assignment was based on the FSS (FSS ≥ 36 points: PCS_fmc_; FSS < 36 points: PCS_fmc_). Exclusion criteria were known neurological/psychiatric diseases prior to the SARS-CoV-2 infection, known hemodynamically relevant carotid artery stenosis or cerebral pathologies, including strokes, detected on structural MRI and not attributable to the SARS-CoV-2 infection. In these cases, patient data were retrospectively excluded from the analysis. Further exclusion criteria included a cognitive or cardiopulmonary inability to perform breath-hold periods of 9 s, pregnancy, and general MRI contraindications. 

#### 2.1.2. Control Persons Previously Infected with SARS-CoV-2 without PCS

Healthy controls were recruited by questioning the relatives of the patients enrolled in the study and through public announcements. Inclusion criteria were an age of 18–50 years and a history of SARS-CoV-2 infection confirmed by polymerase chain reaction (PCR). Analogous to the patient subgroup, exclusion criteria were known neurological/psychiatric diseases prior to the SARS-CoV-2 infection, known as hemodynamically relevant carotid artery stenosis, cerebral pathology detected on structural MRI, pregnancy, the inability to perform breath-hold periods of 9 s and general MRI contraindications. Additionally, subjects were excused if they exhibited any notable symptoms associated with PCS (fatigue, headache, muscle weakness/muscle pain, anxiety/depression, memory/concentration disorders, smell/taste disorders, sleep disturbances, dizziness) subsequent to the SARS-CoV-2 infection.

### 2.2. MRI Data Acquisition

All MR images were acquired on a 3 T MR scanner (Magnetom PrismaFit, Siemens, Erlangen, Germany) using a standard 20-channel head coil. A standardized MRI protocol was performed, including bh-fMRI, as described in detail below, and for the following anatomical sequences: T2-FLAIR, 3D T1-MPRAGE, T2*-weighted images, and TOF-angiography to detect secondary cerebral pathologies which were the exclusion criteria for this study and to quantify white matter changes using the Fazekas score [33].

The fMRI data were acquired using T2*-weighted EPI sequences with the following parameters: TR = 3000 ms, TE = 36 ms, matrix 96 × 96, slice thickness 3 mm, 40 slices in interleaved ascending order, FOV = 245 mm, resolution 2.6 × 2.6 × 3.0 mm^3^, echo spacing: 0.58 ms, TA 9:03 min, and 181 measurements. 

The breath-hold task involved 60 s of normal breathing, followed by 7 repetitive cycles, each consisting of 9 s of end-expiratory breath-holding and 60 s of normal breathing. Respiratory instructions were presented visually via a wall-mounted display using a mirror fixed to the head coil. The instructions were “breathe normally” (60 s), “breathe out” (3 s), and “do not breathe” (9 s). Presentation V20.1 (Neurobehavioral Systems, Berkeley, CA, USA) was used to present scanner-triggered stimuli. To verify the compliance, patients’ respiratory movements during the bh-fMRI task were measured using a pneumatic abdominal belt.

### 2.3. Bh-fMRI Data Processing and Analysis

**Figure 1:**
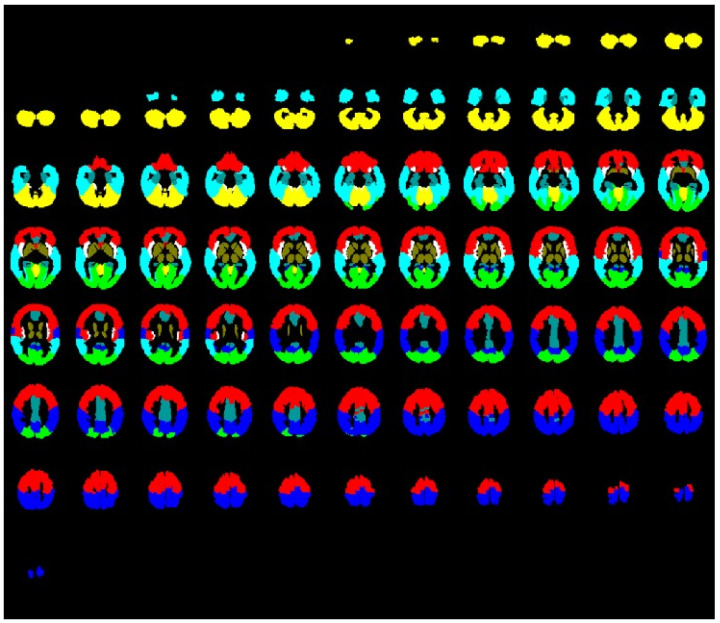
Evaluated regions of interest: global gray matter (all colored tissue), frontal lobe (red), occipital lobe (green), parietal lobe (dark blue), temporal lobe (light blue), limbic system (turquoise), basal ganglia and thalamus (brown), and cerebellum (yellow).

The preprocessing of bh-fMRI data was performed using Statistical Parameter Mapping (SPM12) (https://www.fil.ion.ucl.ac.uk/spm/) (accessed on 30 June 2023), running on MATLAB (R2018b (The MathWorks, Inc., Natick, MA; http://www.mathworks.com) (accessed on 30 June 2023). The DICOM images were converted to NIfTI (Neuroimaging Informatics Technology Initiative) and slice-timing-corrected to compensate for the different image acquisition times, which were realigned to correct for patient head motion, normalized to standard MNI space, segmented into 8 ROIs (global gray matter, frontal lobe, occipital lobe, parietal lobe, temporal lobe, limbic system, basal ganglia and thalamus and cerebellum) (see Figure 1), and spatially smoothed with a Gaussian kernel of 8 mm FWHM. Further data processing was performed using in-house scripts written in MATLAB.

The signal time courses were averaged over the 7 time periods. The percentage of signal change was calculated from the raw data relative to the baseline level, which was seen 42–60 s after the start of the breath-hold period. The time to peak (TTP) of the signal maximum was calculated. The mean signal change over the time period TTP ± 3 s was calculated and considered as CVR. 

### 2.4. Statistical Analysis

All statistical analyses were performed with SPSS Statistics (IBM Corp. Released 2021. IBM SPSS Statistics for Windows, version 28.0. IBM Corp: Armonk, NY, USA). 

First, propensity score matching (PSM) was performed between the patients with PCS and healthy controls to control for the confounding variables of age, sex, and time since infection. Only propensity score-matched data sets were included in the first part of the further analysis.

The percentage signal change and the TTP in each ROI were compared between the PCS_fmc_ subgroup, the PCS_mn_ subgroup, and the healthy control group using one-way analysis of covariance (ANCOVA) while correcting for age, time since infection, Fazekas score, and sex. Bootstrapping with a sample size of 1000 was used to estimate the variability and significance. 

In the second part of the study, we examined the correlation between white matter changes and the CVR in all PCS patients. Healthy controls were not included because white matter lesions in healthy controls were not attributed to the previous SARS-CoV-2 infection. As white matter changes are nonspecific and more common with increasing age, a partial correlation between the global CVR/TTP and the Fazekas score was calculated using age as a control variable. Bootstrapping was performed with a sample size of 1000.

Tests of the 18 a priori hypotheses were performed using Bonferroni-adjusted alpha levels of 0.003 per test (0.05/18).

## 3. Results

### 3.1. Patients

General patient data are presented in Table 1. A total of 44 individuals were examined (16 patients with PCS_fmc_, 11 patients with PCS_mn_, and 17 healthy subjects). One data set had to be excluded due to technical issues during data acquisition, and another data set due to incorrect performance of the bh-task. After PSM, 8 patients were excluded and 8 patients with PCS_fmc_, 8 patients with PCS_mn_, and 17 healthy subjects were included in further analysis (see Table 1).

### 3.2. Regional Differences in the Percentage Signal Change

**Figure 2:**
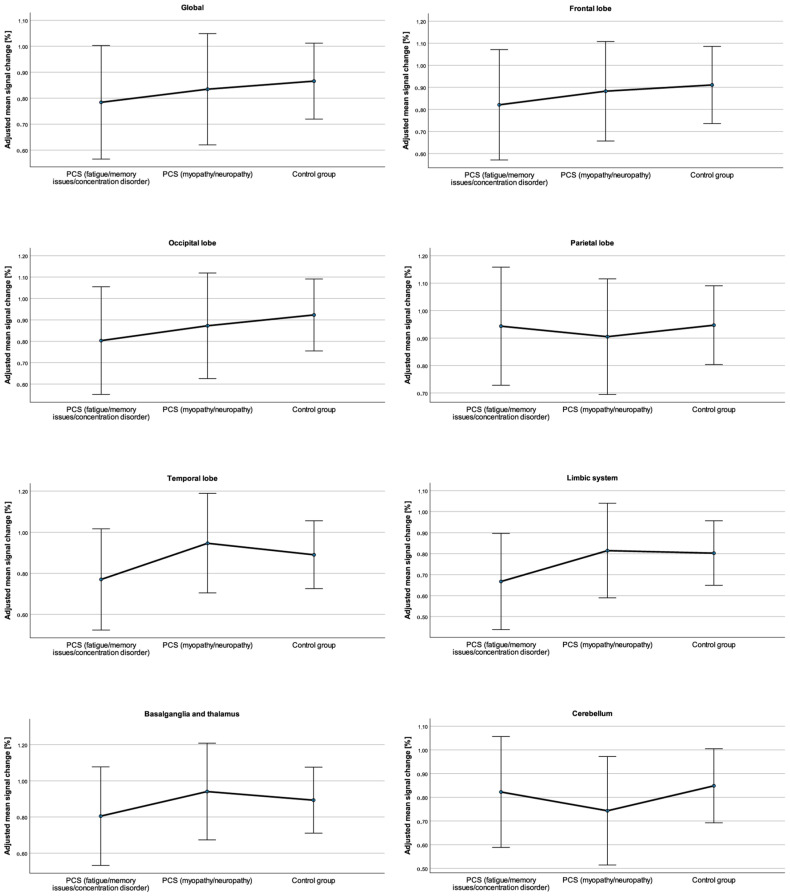
Global and regional mean CVR values and 95% confidence intervals adjusted for age, time since infection, and Fazekas score in patients with post-COVID-19 syndrome (PCS) and leading symptoms of fatigue/memory issues/concentration disorder, patients with PCS and leading symptoms of myopathy/neuropathy, and healthy controls.

The unadjusted global mean CVR values were slightly lower in the PCS_fmc_ subgroup (0.83 ± 0.34%) than in the PCS_mn_ subgroup (0.84 ± 0.22%) and in the control group (0.92 ± 0.27%) (see Table 2). After adjustment for age, time since infection, the Fazekas score, and the global CVR values were also slightly lower in the PCS_fmc_ subgroup (0.78 ± 0.11%) and in the PCS_mn_ subgroup (0.84 ± 0.10%) than in the control group (0.87 ± 0.07%) (see Table 2 and Figure 2). The observed CVR differences between the subgroups after adjustment for age, time since infection, and Fazekas score were not statistically significant: *F*(2, 25) = 0.21, *p* = 0.81, and partial η^2^ = 0.02. There was no significant interaction between subgroup assignment and sex: *F*(2, 25) = 0.19, *p* = 0.83, and partial η^2^ = 0.02.

The unadjusted and adjusted mean CVR values of each ROI are shown in Table 2. After adjustment for age, time since infection and Fazekas score, statistically significant differences in mean CVR were not found between the PCS subgroups and the healthy controls in any ROI: frontal lobe, *F*(2, 25) = 0.58, *p* = 0.57, partial η^2^ = 0.05; occipital lobe, *F*(2, 25) = 0.34, *p* = 0.71, partial η^2^ = 0.03; parietal lobe, *F*(2, 25) = 0.56, *p* = 0.95, partial η^2^ = 0.00; temporal lobe *F*(2, 25) = 0.57, *p* = 0.57, partial η^2^ = 0.04; limbic system *F*(2, 25) = 0.60, *p* = 0.56, partial η^2^ = 0.05; basal ganglia and thalamus *F*(2, 25) = 0.27, *p* = 0.77, partial η^2^ = 0.02; and cerebellum *F*(2, 25) = 0.29, *p* = 0.75, partial η^2^ = 0.02. No significant interactions between the subgroup assignments and sex were observed: frontal lobe, *F*(2, 25) = 0.29, *p* = 0.75, partial η^2^ = 0.02; occipital lobe, *F*(2, 25) = 0.20, *p* = 0.81, partial η^2^ = 0.02; parietal lobe, *F*(2, 25) = 0.76, *p* = 0.48, partial η^2^ = 0.06; temporal lobe *F*(2, 25) = 0.30, *p* = 0.75, partial η^2^ = 0.02; limbic system *F*(2, 25) = 0.10, *p* = 0.90, partial η^2^ = 0.01; basal ganglia and thalamus *F*(2, 25) = 0.04, *p* = 0.97, partial η^2^ = 0.00; and cerebellum *F*(2, 25) = 0.01 *p* = 1.00, partial η^2^ = 0.00.

### 3.3. Regional Differences in the TTP

**Figure 3:**
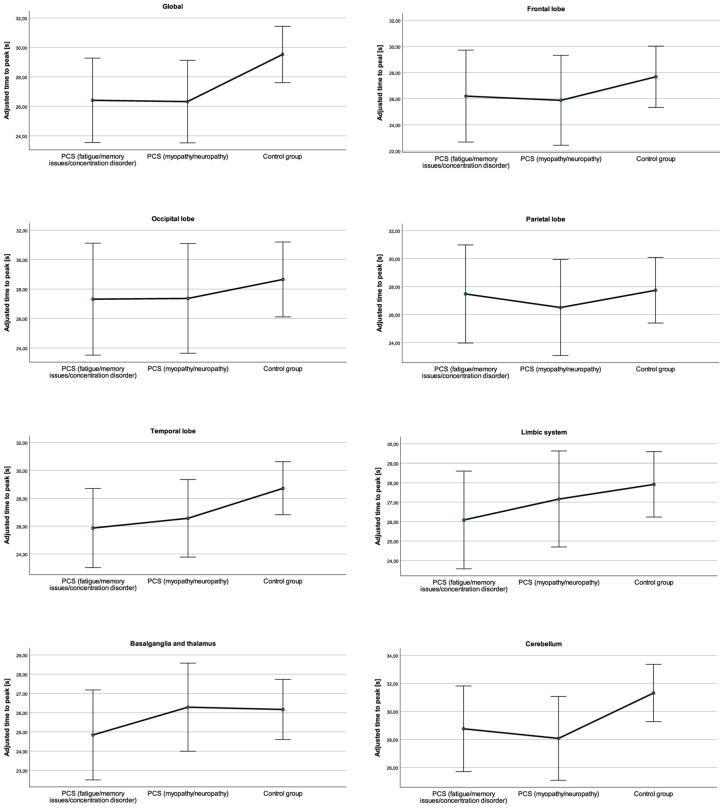
Global and regional time to peak values and 95% confidence intervals adjusted for age, time since infection, and Fazekas score in patients with post-COVID-19 syndrome (PCS) and leading symptoms of fatigue/memory issues/concentration disorder, patients with PCS and leading symptoms of myopathy/neuropathy, and healthy controls.

The unadjusted mean TTP was smaller in the PCS_fmc_ subgroup (27.20 ± 3.19 s) and in the PCS_mn_ subgroup (26.10 ± 2.51 s) than in the control group (29.08 ± 3.82 s) (see Table 2). After adjustment, the global mean TTP was also smaller in the PCS_fmc_ subgroup (26.41 ± 1.39 s) and in the PCS_mn_ subgroup (26.32 ± 1.36 s) than in the control group (29.52 ± 0.93 s) (see Table 2 and Figure 3), but the differences in the TTP after adjustment for age, time since infection and Fazekas score, were not statistically significant, *F*(2, 25) = 2.74, *p* = 0.08, partial η^2^ = 0.18. There was no significant interaction between subgroup assignment and sex that was observed: *F*(2, 25) = 1.81, *p* = 0.19, and partial η^2^ = 0.13.

In the ROI analysis, there were also trends towards a smaller TTP in PCS patients than in healthy controls (see Table 2 and Figure 3). However, after adjustment for age, time since infection, Fazekas score and sex, these differences were not significant as follows: frontal lobe, *F*(2, 25) = 0.50, *p* = 0.61, partial η^2^ = 0.04; occipital lobe, *F*(2, 25) = 0.27, *p* = 0.77, partial η^2^ = 0.02; parietal lobe, *F*(2, 25) = 0.18, *p* = 0.84, partial η^2^ = 0.01; temporal lobe *F*(2, 25) = 1.83, *p* = 0.18, partial η^2^ = 0.13; limbic system *F*(2, 25) = 0.80, *p* = 0.46, partial η^2^ = 0.06; basal ganglia and thalamus *F*(2, 25) = 0.56, *p* = 0.58, partial η^2^ = 0.04; and cerebellum *F*(2, 25) = 2.06, *p* = 0.15, partial η^2^ = 0.14. There was no significant interaction between subgroup assignment and sex: frontal lobe, *F*(2, 25) = 1.10, *p* = 0.35, partial η^2^ = 0.08; occipital lobe, *F*(2, 25) = 0.58, *p* = 0.57, partial η^2^ = 0.04; parietal lobe, *F*(2, 25) = 0.49, *p* = 0.62, partial η^2^ = 0.04; temporal lobe *F*(2, 25) = 1.72, *p* = 0.20, partial η^2^ = 0.12; limbic system *F*(2, 25) = 1.57, *p* = 0.23, partial η^2^ = 0.11; basal ganglia and thalamus *F*(2, 25) = 1.01, *p* = 0.39, partial η^2^ = 0.08; and cerebellum *F*(2, 25) = 1.28, *p* = 0.30, partial η^2^ = 0.93.

### 3.4. Correlation between CVR and White Matter Changes

After adjustment for age, there was a very slight trend toward lower global CVR values in PCS patients with more white matter changes, but this correlation was weak and not significant (*r* = −0.10; *p* = 0.65, 95% CI: −0.49–0.30). After adjustment for age, the global TTP tended to not be significantly smaller in patients with more white matter changes (*r* = −0.24; *p* = 0.26, 95% CI: −0.51–0.11). 

## 4. Discussion

The aim of this study was to compare the global and regional CVR between patients with PCS_fmc_, patients with PCS_mn_, and healthy controls using bh-fMRI.

To date, the diagnosis of PCS remains a diagnosis of exclusion [2], making it challenging for clinicians and unsatisfactory for patients. In this study, we aimed to investigate whether CVR measured with bh-fMRI could be a complementary marker for the diagnosis of PCS, which could influence patient management and therapy. The CVR can change over time in patients with different neurological manifestations and may, therefore, be a biomarker for disease progression and treatment response [6,19]. Furthermore, the exact pathomechanism behind PCS is an ongoing topic of research [5,6]. Therefore, an additional motivation for this study was to investigate whether vascular damage is the most important pathomechanism, which may also influence long-term treatment options. Treatment options that affect endothelial function, such as beta-blockers, angiotensin-converting enzyme inhibitors, angiotensin receptor blockers, and statins, have already been discussed [11]. 

In line with the findings of Callen et al. [15], patients with PCS in this pilot study showed a slight trend toward lower CVR. However, no significant global or regional differences in the percentage signal change were observed after adjusting for time since infection, age, sex, and white matter lesions. 

There were no significant global or regional differences in TTP after adjustment for time since infection, age, sex, and white matter lesions. It was noticeable that the patients with PCS tended to show a reduced TTP (approximately 3 s). A possible explanation could be the pulmonary impairment of PCS patients as part of the SARS-CoV-2 infection. Pulmonary diseases have an impact on the change in PaCO_2_ [16]. A limited pulmonary reserve in patients with PCS could result in a different baseline PaCO_2_ and a faster increase in the PaCO_2,_ and, therefore, a lower TTP.

In this study, there was no significant correlation between the CVR and white matter changes. One reason might be that it is unclear whether the observed white matter lesions were due to SARS-CoV-2 infection, although we controlled for age to account for age-related microvascular lesions. However, it is likely that most of the observed lesions were nonspecific. Another possible explanation is that structural vascular changes that induce anatomical white matter changes are expected to occur later than functional changes [6] and may not yet be evident in PCS patients. According to previous studies, neurological symptoms are not associated with white matter lesions [34,35].

Several previous studies investigating the CVR with TCCD in patients with acute SARS-CoV-2 infection described significantly lower CVR in PCS patients than in control groups [19,20]. Nandadeva et al. reported contrary results and found no significant differences between PCS patients and healthy controls [21].

Compared with the study of Callen et al. and most of the other studies using TCCD, the present study involved a longer time period between the SARS-CoV-2 infection and the acquisition of MRI data [6,15]. This might be one reason why no significant CVR differences were reproduced. Marcic et al., who compared the CVR between neurological PCS patients with a relatively long time period since infection (approximately 10 months) and controls, found significant differences in the CVR between the two groups [18]. However, in this study, the time period between SARS-CoV-2 infection and MRI was even longer (approximately 20 months).

This study has limitations. One limitation was the small sample size, which may account for the lack of significance of the results. To overcome this limitation, we aimed to provide more robust estimates of the CVR using propensity score matching to reduce bias and using bootstrapping resampling to provide confidence intervals. Another issue was that some PCS patients presented with symptoms of both subgroups, which could reduce the expected effect of a lower CVR value in the PCS_fmc_ subgroup presenting with predominantly CNS symptoms. Another limitation is that the most severely affected patients, who might have shown the strongest effects, could not be included in the study because they were unable to undergo MRI measurements with breath-hold tasks. [6,35]

The bh-fMRI technique is a promising method, which has already found clinical application in macrovascular diseases, such as Moyamoya angiopathy, where it shows comparable results to the diagnostic gold standard [^15^O] water PET [17,23,24,36,37]. It is possible that the method bh-fMRI is particularly unsuitable for PCS patients as it requires patient cooperation and the absence of severe preexisting lung disease [16]. In addition to bh-fMRI, hypercapnia-triggered fMRI with the CO_2_ inhalation challenge is an alternative method to measure the CVR [13,16,38,39]. While TCCD is limited to the assessment of large arteries, both MRI-based methods allow for the assessment of global and regional CVR with high spatial resolution [13]. Bh-fMRI does not require hardware to control hypercapnia [13]. However, a major advantage of hypercapnia-triggered fMRI with the CO_2_ inhalation challenge using a custom-built breathing circuit (e.g., RespirAct, Thornhill, Toronto, Canada [40,41,42]) is that it is less dependent on patient cooperation and provides a standardized and controlled hypercapnic stimulus, allowing for more standardized assessments and probably more reliable data [13,16,22]. 

This study was a pilot study. Further studies, e.g., using hypercapnia-triggered fMRI with CO_2_ inhalation challenge in larger cohorts, could address this issue to reevaluate whether significant differences between PCS patients and healthy controls can be detected.

Finally, the neurological manifestations of SARS-CoV-2 have been attributed to multiple overlapping pathomechanisms [6,43]. The small effects observed in this study suggest that endothelial damage, as measured by the use of bh-fMRI, is not the only pathogenetic mechanism leading to neurological manifestations of PCS. 

## 5. Conclusions

In this pilot study, no significant differences in CVR between patients with neurological PCS and controls were found. This suggests that endothelial damage is not the sole pathomechanism of neurological PCS. Further studies in larger cohorts are needed.

## Figures and Tables

**Table 1:** General patient data.

	Patients with PCS_fmc_^1^		Patients with PCS_mn_^2^		Healthy Subjects with Previous SARS-CoV-2 Infection	
	Before PSM ^3^	After PSM ^3^	Before PSM ^3^	After PSM ^3^	Before PSM ^3^	After PSM ^3^
**Number of persons**	14	9	11	8	17	17
**Female/male ratio**	6.0:1	3.5:1	1.8:1	1.7:1	2.4:1	2.4:1
**Mean age [years] (SD)**	38.9 (9.9)	38.7 (2.7)	41.1 (7.6)	37.8 (2.5)	32.9 (6.3)	33.9 (2.3)
**Time after SARS-CoV-2 infection [months] (SD)**	22.6 (8.4)	21.1 (2.9)	21.4 (10.9)	19.8 (4.0)	19.7 (2.4)	19.7 (2.4)
**Fazekas score (SD)**	0.2 (0.1)	0.2 (0.2)	0.7 (0.2)	0.6 (0.3)	0.2 (0.1)	0.2 (0.1)

^1^ PCS_fmc_ = Post-COVID-19 syndrome with leading symptoms of fatigue/memory issues/concentration disorder. ^2^ PCS_mn_ = Post-COVID-19 syndrome with leading symptoms of myopathy/neuropathy. ^3^ PSM = propensity score matching.

**Table 2:** Global and regional unadjusted and adjusted differences in signal change and time to peak between patients with post-COVID-19 syndrome and healthy controls.

		Unadjusted		Adjusted		Unadjusted		Adjusted	
		Signal Change [%] (Mean ± SD)	CI	Signal Change [%] (Mean ± SD)	CI	Time to Peak [s] (Mean ± SD)	CI	Time to Peak [s] (Mean ± SD)	CI
**Global**	PCS_fmc_^1^	0.83 ± 0.32	0.63–1.03	0.78 ± 0.11	0.60–1.01	27.20 ± 3.19	25.12–29.12	26.41 ± 1.39	24.91–29.70
	PCS_mn_^2^	0.84 ± 0.22	0.71–1.01	0.84 ± 0.10	0.62–1.06	26.10 ± 2.51	24.40–27.86	26.32 ± 1.36	23.10–28.70
	Control group	0.92 ± 0.25	0.82–1.04	0.87 ± 0.07	0.75–1.02	29.08 ± 3.82	27.41–30.97	29.52 ± 0.93	26.79–31.85
**Frontal lobe**	PCS_fmc_^1^	0.77 ± 0.45	0.46–1.03	0.77 ± 0.13	0.53–0.99	26.97 ± 3.25	25.12–29.12	26.16 ± 1.71	24.46–29.61
	PCS_mn_^2^	0.93 ± 0.17	0.81–1.05	0.91 ± 0.12	0.70–1.11	26.06 ± 2.52	24.40–27.86	25.87 ± 1.67	22.31–28.55
	Control group	0.97 ± 0.23	0.86–1.09	0.92 ± 0.08	0.77–1.14	27.69 ± 5.53	25.00–30.18	27.68 ± 1.17	23.84–31.30
**Occipital lobe**	PCS_fmc_^1^	0.89 ± 0.38	0.65–1.13	0.80 ± 0.12	0.63–1.09	27.45 ± 3.06	25.58–29.70	27.32 ± 1.85	24.80–30.14
	PCS_mn_^2^	0.87 ± 0.20	0.73–1.01	0.87 ± 0.12	0.63–1.07	27.78 ± 3.31	25.60–30.26	27.37 ± 1.81	22.91–30.55
	Control group	0.99 ± 0.30	0.86–1.14	0.92 ± 0.08	0.80–1.08	28.46 ± 5.75	25.64–30.87	28.66 ± 1.23	24.32–31.99
**Parietal lobe**	PCS_fmc_^1^	1.01 ± 0.27	0.84–1.19	0.94 ± 0.10	0.82–1.21	27.57 ± 2.67	25.89–29.37	27.48 ± 1.70	25.36–30.14
	PCS_mn_^2^	0.94 ± 0.15	0.83–1.04	0.91 ± 0.02	0.63–1.06	26.51 ± 2.44	24.90–28.37	26.50 ± 1.67	22.75–29.29
	Control group	1.02 ± 0.34	0.85–1.18	0.95 ± 0.07	0.78–1.12	27.71 ± 5.50	24.76–30.13	27.74± 1.14	22.91–31.49
**Temporal lobe**	PCS_fmc_^1^	0.86 ± 0.36	0.65–1.10	0.77 ± 0.12	0.56–1.06	26.60 ± 3.73	24.63–29.36	25.87 ± 1.38	24.22–29.08
	PCS_mn_^2^	0.94 ± 0.28	0.78–1.17	0.95 ± 0.12	0.71–1.24	26.25 ± 2.18	25.00–27.97	26.56 ± 1.36	24.16–28.82
	Control group	0.94 ± 0.25	0.82–1.06	0.89 ± 0.08	0.75–1.08	28.25 ± 3.67	26.91–30.40	28.72± 0.92	26.26–31.10
**Limbic system**	PCS_fmc_^1^	0.73 ± 0.35	0.52–0.96	0.67 ± 0.11	0.46–0.92	27.13 ± 3.67	25.08–29.85	26.09 ± 1.22	24.66–29.19
	PCS_mn_^2^	0.84 ± 0.22	0.72–1.03	0.82 ± 0.11	0.58–1.11	26.32 ± 3.52	24.81–28.35	27.16 ± 1.20	24.76–29.39
	Control group	0.85 ± 0.24	0.75–0.96	0.80 ± 0.07	0.66–0.99	28.16 ± 2.75	27.02–29.57	27.92± 0.82	26.18–29.25
**Basal ganglia and thalamus**	PCS_fmc_^1^	0.82 ± 0.36	0.60–1.06	0.81 ± 0.13	0.62–1.03	25.40 ± 3.47	23.93–27.95	24.84 ± 1.14	23.76–27.04
	PCS_mn_^2^	1.01 ± 0.34	0.77–1.28	0.94 ± 0.13	0.67–1.33	25.61 ± 2.12	24.24–28.35	26.28 ± 1.11	24.21–28.00
	Control group	0.93 ± 0.30	0.79–1.09	0.89 ± 0.09	0.75–1.08	26.03 ± 2.46	25.03–27.21	26.16± 0.76	25.03–27.13
**Cerebellum**	PCS_fmc_^1^	0.87 ± 0.27	0.59–0.97	0.82 ± 0.11	0.61–1.05	28.93 ± 4.00	26.67–31.63	28.76 ± 1.49	27.08–31.64
	PCS_mn_^2^	0.90 ± 0.26	0.80–1.02	0.74 ± 0.11	0.53–1.01	28.16 ± 3.49	25.72–30.51	28.07 ± 1.45	24.81–30.71
	Control group	0.85 ± 0.27	0.77–0.94	0.85 ± 0.08	0.72–1.02	30.53 ± 3.74	28.82–32.27	31.31± 0.99	28.99–33.34

^1^ PCS_fmc_ = Post-COVID-19 syndrome with leading symptoms of fatigue/memory issues/concentration disorder. ^2^ PCS_mn_ = Post-COVID-19 syndrome with leading symptoms of myopathy/neuropathy.

## Data Availability

In order to safeguard the confidentiality of the participants, the data pertaining to this study are currently withheld from public access. The data can be shared upon request.
